# Contributing Factors for Acute Illness/Injury from Childhood Pesticide Exposure in North Carolina, USA, 2007–2013

**DOI:** 10.3390/toxics4010004

**Published:** 2016-02-02

**Authors:** Nirmalla Barros, Ricky Langley, Wayne Buhler, Kelly Brantham

**Affiliations:** 1 Division of Public Health, North Carolina Department of Health and Human Services, 1912 Mail Service Center, Raleigh, NC 27699-1912, USA; rick.langley@dhhs.nc.gov (R.L.); kelly.brantham@dhhs.nc.gov (K.B.); 2 Department of Horticultural Science, North Carolina State University, Campus Box 7609, Raleigh, NC 27695, USA; wbuhler@ncsu.edu

**Keywords:** pesticide, children, exposure, illness, injury

## Abstract

Between 2007 and 2013, there were 685 events with evidence of a relationship between pesticide exposure and acute illness/injury among persons less than 18 years old in North Carolina (United States). Median age of children affected was 4.3 years (range: 0.2–17.9). Distribution by gender was similar across all age groups. One fatality and four high severity events were observed. The greatest proportion (42%) of events had ocular exposures, followed by dermal (25%) and inhalation (18%) exposures. When more than one route of exposure occurred, dermal and ocular routes were the most common (46%). Almost all events took place indoors and 32 events involved contact with pets. Insecticides (53%) and insect repellants (31%) were the most frequent agents contributing to these events. Manual application of pesticides contributed to the greatest number of events (25%), while application through a pressurized can and use of a trigger pump were involved in 21% and 15% of events, respectively. Additional contributors were due to inappropriate storage of pesticides and improper use of the pesticide. These contributing factors can be removed or minimized if pesticides are stored outside the residence or out of the reach of children and pets, and adequate ventilation is ensured whenever pesticides are applied.

## 1. Introduction

A pesticide is a substance or mixture of substances that is applied to prevent, destroy, repel, or mitigate a pest [1]. Pesticides can be classified based on the pest they control (*i.e.*, herbicides for weeds, insecticides for insects) or their chemical class (*i.e.*, organophosphates, pyrethroid, organochlorine, *etc.*). The United States Environmental Protection Agency estimated that more than 1.1 billion pounds of conventional and other (such as sulfur and petroleum oil) pesticides (excludes specialty biocides, chlorine/hypochlorites used in water treatment, and wood preservatives) were used in the U.S. in 2007, with the greatest proportion being used for herbicides (47%) [2]. According to the United States Department of Agriculture’s 2012 Census of Agriculture, nearly 7.5 million acres of farmland in the southeastern state of North Carolina (United States) were treated with pesticides to kill insects, weeds, nematodes, and diseases [3].

Between 2006 and 2010, a total of 650,682 calls were made to poison centers in the United States related to exposure to pesticides; slightly more than 40% of these calls affected an individual five years of age or younger [4]. From 1999 to 2008, there were 234 deaths due to pesticide poisonings (averaging 23 deaths per year) [4]; five percent of these deaths were among children aged 19 years or younger [5]. From 1990 to 1993, 29% of hospitalizations for pesticide poisoning were among children less than 14 years in North Carolina [6].

Compared with adults, children may be more vulnerable to particular risks from exposure to pesticides and their toxic effects [7,8,9]. This vulnerability can be due to a number of factors, including differences in physiology, behavior, and environmental conditions [7,9]. Development of the brain occurs early in life at rigid time frames and in different regions, making the brain vulnerable to any influences deterring this development [8]. The potential for the occurrence of exposures of pesticides during critical stages of early neurological development may impede further development and may lead to lifetime morbidity [7]. In addition, a child’s distinctive behavior (e.g., crawling on floors) and their environmental conditions (e.g., pesticide treatment of floors or surfaces) when engaging in this behavior can increase their exposure. Children can also be exposed through more than one route to the same substance as well as through contact with different substances (e.g., household, outdoors) by virtue of their behavior of object/surface-to-skin/mouth responses [8]. This behavior is often exhibited as frequent contact with objects/surfaces and then potential exposure transfer of these substances through contact with the body (e.g., mouth, skin) [8,9].

In an effort to monitor pesticide-related illnesses and injuries, the state of North Carolina passed a mandatory reporting rule in 2006. This rule requires clinical providers to report acute pesticide-related illness or injury within 48 h after the illness or injury is diagnosed, and immediately if the acute pesticide-related illness or injury results in death [10].

To provide information that is crucial for prevention and control of pesticide-related illness or injury among high-risk populations, such as children, this study aimed to describe the occurrence of childhood pesticide exposures and subsequent acute illness or injury in North Carolina. Further aims of this study were to characterize contributing factors for exposure, including source, manner of contact with pesticides, and their reported toxic effects.

## 2. Methods

### 2.1. Data Sources

The North Carolina Division of Public Health’s Pesticide Incident Surveillance Program (NC PISP) collects information about reports of pesticide exposure and acute illness or injury from clinical care facilities in the state. Clinicians submit these reports to the Carolinas Poison Center (CPC). The CPC then submits them to the NC PISP [11]. These reports include contact information and characteristics (age, race/ethnicity, gender, and occupation) of the exposed person(s); the physical location of the affected person(s) at the time of the exposure to the pesticide(s); the name of the pesticide(s) of interest; and the contact information of the reporting physician or medical facility.

In addition to events reported from clinical care facilities, information about exposure to pesticides and acute illness or injury, which were reported to the NC PISP, were obtained from inquiries made to the CPC by the general public and reports from local and state government agencies in North Carolina. Data used for this study were retrieved from the NC PISP’s reports and inquiries.

### 2.2. Classification of Pesticide Exposure and Acute Illness/Injury Event

Events were investigated by the NC PISP to obtain additional exposure and acute illness or injury information for the affected person(s) if they took place at a workplace. If they occurred at a non-occupational setting, they were investigated if the illness or injury was severe, which was based on signs and symptoms, type of medical care sought (e.g., emergency department visit, hospitalization), and whether lost time from work or usual activities took place. These non-occupational events were also investigated if a death occurred, the exposure took place at a school, the pesticide was sprayed over a public area, the exposure affected more than two persons, and if the exposure was due to drift (*i.e.*, movement of the pesticide away from the application site).

Additional information about the event was gathered by an interview of the affected person or a guardian, if the affected person was less than 18 years of age at the time of the event. In addition, medical records were requested from the health care facility if the exposed person sought medical care after the exposure occurred.

Based on information gathered from interviews and medical records, the program identified and classified events as “confirmed” if sufficient evidence was available to support a causal relationship between the pesticide exposure(s) and acute illness or injury. The event was also given a severity score based on the severity of the health effect(s) (e.g., death, injury, illness, signs and symptoms), and type of medical care sought. Events of high severity were severe enough to be considered potentially life-threatening, which typically require treatment and time away from work. Events of moderate severity resulted in less severe illness or injury, and the individual was typically able to return to usual activities without any disability. In low severity events, affected individuals often exhibited signs and symptoms such as headache, fatigue, or eye, skin, or respiratory irritation that tends to be resolved without treatment. These criteria (*i.e.*, confirmation status and severity score) were developed by the United States National Institute for Occupational Safety and Health’s SENSOR (Sentinel Event Notification System for Occupational Risk)-Pesticides Program (Centers for Disease Control and Prevention) [12].

### 2.3. Sample Selection

The criteria for inclusion in this study were: (1) the occurrence of the event in North Carolina; (2) the classification of the event as having sufficient evidence to confirm a causal relationship between the pesticide exposure and acute illness, injury, or death; (3) the affected person was less than 18 years old at the time of the event (*i.e.*, childhood); and (4) the event was reported between 2007 (events reported before this time were few and inconsistently reported) and 2013 (events reported after this time had not been given a classification at the time of preparation of this manuscript).

### 2.4. Descriptive Analysis

Analyses were performed to summarize which age groups tended to have the most severe illness or injury following the exposure event, the location where exposures most likely occurred, the type of contact to the agent (e.g., spray, spill), and the time of year when exposures peaked among this population. Other contributing factors such as activity and equipment used at the time of the exposure event were also examined.

## 3. Results

**Figure 1:**
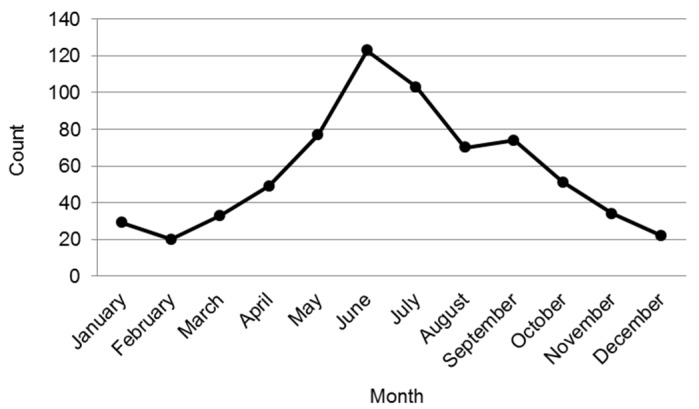
Distribution of events in childhood reported by month, 2007–2013.

From 2007 to 2013, there were 685 reported events that had sufficient evidence of a relationship between their pesticide exposure and subsequent acute illness, injury, or death among those aged less than 18 years at the time of the event in North Carolina. Almost all of the events (99%) were reported through the CPC. Reported events peaked in the warmer month of June (*n* = 123) (Figure 1). The fewest number of events was reported in the colder months of February (*n* = 20) and December (*n* = 22).

### 3.1. Sample Characteristics

The median age of the affected individuals was 4.3 years (range: 0.2–17.9 years). A little more than half (55%) of these events occurred among children between one and five years old at the time of the exposure (Table 1). Slightly more than half (56%) were among males. The distribution by gender was similar across all age groups. Information about race and Hispanic ethnicity was not available for more than 90% of affected individuals.

**Table 1:** Distribution of events in childhood reported by age group and gender.

Age Group (Year)	Total		Female		Male	
	No.	% of Age Group Total	No.	% of Age Group Total	No.	% of Age Group Total
<1	11	2	5	1	6	1
1–5	381	55	172	25	209	30
6–12	164	24	64	9	100	15
13–<18	129	19	60	9	69	10
Total	685	100	301	44	384	56

### 3.2. Health Effects

From 2007 to 2013, there was one male child (eight years of age) who was hospitalized and then died from his pesticide exposure (Table 2). The child drank a herbicide containing paraquat. The substance was placed into a (soda) bottle and left by a garage window by the individual’s brother, who got the substance from a certified pesticide applicator. The child died about two weeks later after ingestion.

There were four high severity pesticide events: one that affected a young child’s (aged less than a year old) respiratory and nervous/sensory systems; and three affecting older children’s (13 to 15 years old) cardiovascular, gastrointestinal, nervous/sensory, and respiratory systems. More than 90% of the reports were of low severity; the greatest proportion of these events involved eye irritation.

**Table 2:** Distribution of events in childhood reported by severity of illness/injury and age group.

Severity	No. (%)
Death	1 (<1)
<1 year	0 (0)
1–5 years	0 (0)
6–12 years	1 (100)
13–<18 years	0 (0)
High	4 (<1)
<1 year	1 (25)
1–5 years	0 (0)
6–12 years	0 (0)
13–<18 years	3 (75)
Moderate	18 (3)
<1 year	0 (0)
1–5 years	11 (61)
6–12 years	2 (11)
13–<18 years	5 (28)
Low	662 (96)
<1 year	10 (2)
1–5 years	370 (56)
6–12 years	161 (24)
13–<18 years	121 (18)

Among those who sought initial medical care, 259 (38%) contacted the CPC for consultation and were from the general public, and 41 (6%) visited the emergency room. For those who visited an emergency room, two (5%) were aged less than one year old, 25 (61%) were greater than one year old and less than 12 years old, and 14 (34%) were adolescents (*i.e.*, >12 to <18 years).

A small proportion of the affected population had pre-existing conditions, such as allergies (4%), asthma (5%), or multiple chemical sensitivities (<1%) that could have had an effect on the response to their exposure (Table 3).

**Table 3:** Distribution of events in childhood reported by pre-existing condition that could impact response to exposure.

Type of Report	Pre-Existing Condition No. (%)		
	Allergies	Asthma	Multiple Chemical Sensitivities
Clinician reported	1 (<1)	1 (<1)	0 (0)
Exposed individual reported	21 (3)	31 (4)	2 (<1)
Reported by both	2 (<1)	2 (<1)	0 (0)
Pre-existing condition not present	578 (84)	571 (83)	594 (86)
Unknown	83 (12)	80 (11)	89 (13)

### 3.3. Pesticide Exposure

Nearly all pesticide exposure events (98%) were unintentional (Table 4). Six events (1%) were suspected as intentional pesticide exposures, with five of the events involving an insecticide; the affected individuals ranged from 11 to 17.5 years. A small proportion (1%) were related to exposures affecting adolescents (13 to 17 years old) while at work.

**Table 4:** Distribution of events in childhood reported by cause.

Intent/Cause	No. (%)
Intentional	6 (1)
Accidental	670 (98)
Unknown	9 (1)
Non-occupational	678 (99)
Occupational	7 (1)

#### 3.3.1. Route of Exposure

**Figure 2:**
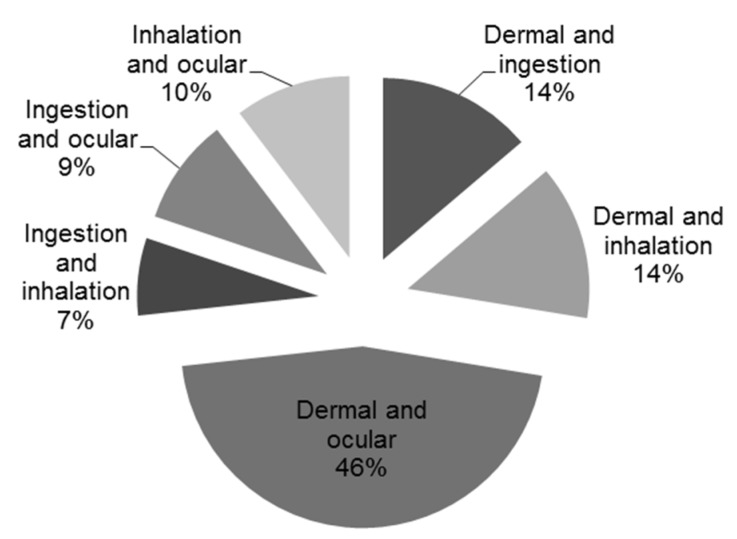
Distribution of events in childhood reported to have exposure by more than one route.

Compared with other routes of exposure, the greatest number of reports (*n* = 321) were exposures through the eye, with the greatest proportion (67%) affecting children between one and five years old (Table 5). The fewest were found to occur through the mouth (*n* = 112). Among those who had exposure by more than one route (*n* = 116), a little less than half (46%) were affected through the eyes and skin (Figure 2).

**Table 5:** Distribution of events in childhood reported by primary route of exposure and age group.

Route ^1^	No. (%)
Dermal	194 (25)
<1 year	3 (2)
1–5 years	89 (46)
6–12 years	59 (30)
13–<18 years	43 (22)
Inhalation	139 (18)
<1 year	6 (4)
1–5 years	42 (30)
6–12 years	51 (37)
13–<18 years	40 (29)
Ingestion	112 (15)
<1 year	1 (1)
1–5 years	84 (75)
6–12 years	8 (7)
13–<18 years	19 (17)
Ocular	321 (42)
<1 year	4 (1)
1–5 years	215 (67)
6–12 years	67 (21)
13–<18 years	35 (11)

^1^ refers to some events reported exposure through more than one route.

#### 3.3.2. Source of Exposure

**Figure 3:**
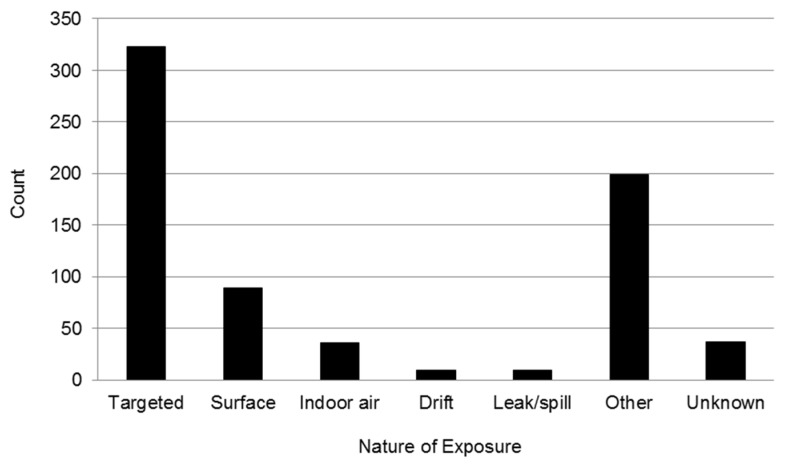
Distribution of events in childhood reported by nature of exposure (some exposures classified into more than one grouping).

Overall, the greatest number of reports involved targeted applications of a pesticide material (*i.e.*, pesticide released at the treatment site and not carried from the target site by air) (Figure 3). These applications can include airborne exposures to individuals moving in the area when treatment is applied and direct projections, ricochet, or blowback by wind. Among the 89 events that occurred from surface applications (*i.e.*, individuals exposed through contact with pesticide residues on treated surfaces or animals or entry into an outdoor treated area), 36% were due to interactions with a pet.

Manual placement of pesticides was reported as the application method contributing to the most number of pesticide exposure events (25%); other main methods included the application from a pressurized can (21%) and the use of a trigger pump (15%) (Table 6). Among the known contributors of pesticide exposure events by accidental causes, the greatest proportion (68%) were due to a pesticide being stored within reach of the child (Table 7). Improper use of the pesticide according to its label was the next main factor (18%) in playing a role in contributing to exposure.

**Table 6:** Distribution of events in childhood by equipment or application method.

Type of Application Method or Equipment	No. (%)
Manual placement	168 (25)
Pressurized can	146 (21)
Trigger pump (or push-pull/compressed air hand sprayer)	102 (15)
Total release fogger or aerosol bomb	38 (6)
More than one type of application equipment used	4 (1)
Other	3 (<1)
Soil injector	2 (<1)
Handheld granular or dust applicator	1 (<1)
Spray line, hand held	1 (<1)
Sprayer, back pack	1 (<1)
Low-pressure ground sprayer	1 (<1)
Not applicable	105 (15)
Unknown	113 (16)

**Table 7:** Distribution of events in childhood reported by contributing factor of accidental causes.

Contributing Factor	No. (%)
Pesticide stored within reach of child/other improper storage	297 (67)
Label violations	81 (18)
No label violation identified but person still exposed/ill	24 (5)
Located in treated area during application	12 (3)
Spill/splash of liquid or dust	12 (3)
Excessive application of pesticide	7 (1)
Drift	6 (1)
Decontamination not adequate or timely	3 (<1)
Early re-entry	2 (<1)
Inadequate ventilation of treated area before re-entry	1 (<1)
Application equipment failure	1 (<1)
Total	446 (100)

Among events with available information about known agents contributing to pesticide exposure, slightly more than half of these reports (53%) were from insecticides (Table 8). For events affecting each age group, the greatest proportion of events was from insecticides followed by insect repellant.

**Table 8:** Distribution of events in childhood reported by pesticide functional class and age group.

Pesticide ^1^	All Events	<1 year	1–5 years	6–12 years	13–<18 years
	No. (%)	No. (%)	No. (%)	No. (%)	No. (%)
Fumigant	4 (1)	0 (0)	1 (<1)	3 (3)	0 (0)
Fungicide	0 (0)	0 (0)	0 (0)	0 (0)	0 (0)
Herbicide	25 (5)	0 (0)	15 (4)	6 (6)	4 (4)
Insecticide	274 (53)	4 (40)	140 (45)	60 (58)	70 (72)
Insect repellant	161 (31)	4 (40)	119 (39)	26 (25)	12 (12)
Rodenticide	8 (2)	0 (0)	8 (3)	0 (0)	0 (0)
Other ^2^	15 (3)	0 (0)	8 3)	4 (4)	3 (3)
Multiple ^3^	29 (5)	2 (10)	14 (5)	5 (5)	8 (8)
Total	516 (100)	10 (100)	305 (100)	104 (100)	97 (100)

^1^ refers to the substance that was grouped into categories by the pest that it aims to control; ^2^ refers to substances that cannot be grouped in the above categories; ^3^ refers to substances that control more than one grouping of pests.

#### 3.3.3. Location of Exposure

Nearly all pesticide exposures that were reported during childhood took place at an indoor setting; almost all (98%) occurred at a private residence (Table 9). There were few reports (1%) that took place on farms.

**Table 9:** Distribution of events in childhood reported by location where event took place.

Location	No. (%)
Indoor	649 (95)
Institution, outside of residence ^1^	6 (<1)
Non-manufacturing commercial facilities ^2^	7 (1)
Private residence	634 (98)
Vehicle	2 (<1)
Outdoor	11 (2)
Farm	10 (91)
Park	1 (9)
Other (site not specified)	15 (2)
Unknown	10 (1)
Total	685 (100)

^1^ schools, residential institution; ^2^ retail and service establishments, pet services.

## 4. Discussion

From 2007 to 2013, there were 685 pesticide exposure events reported to the Carolinas Poison Center among residents of the southeastern state of North Carolina (United States) who were less than 18 years of age at the time of the exposure. Almost all events involved accidental exposures; six events were due to intentional exposures, of which five involved insecticides affecting children ranging from 11 to 17.5 years. There was one reported fatality that took place at the child’s residence where an herbicide containing paraquat was ingested. Death can often occur: within 24 h, if more than 100 milligrams of paraquat is ingested; within 7 days, if between 50 to 100 milligrams is ingested; and within 2 to 4 weeks, if 15 to 40 milligrams is ingested [13].

Overall, pesticide exposure events among children were found to occur most often when an insecticide or insect repellant was applied. Almost all of these events took place indoors in a residence and tended to peak in the warmer months of the year. In comparison, studies focusing on workers found that insecticides and herbicides were the most commonly reported exposures [14,15,16].

In this study, children less than 18 years of age were most often exposed to pesticides through their eyes (42% of reports), followed by dermal exposure (25%), inhalation (18%), and ingestion (15%). Belson *et al.* [17] noted children younger than 6 years, who lived near the state of Texas and the Mexico border between 1997 and 2000, to be exposed more frequently to pesticides through ingestion (86%), followed by dermal exposure (8%), ocular exposure (6%), and inhalation (2%). When we restricted our events to only those affecting children younger than 6 years old in this study, ocular exposures were still the primary route (56%) (dermal and ingestion exposures contributed to 24% and 21% of events, respectively). Among workers, however, dermal exposure, primarily through contact with hands, represented the greatest risk for occupational exposures [15].

This study found 32 reports of pesticide exposure from contact with animals. It has been shown in various studies that pesticide products applied to animals to control various ectoparasites can be transferred to humans, and that the exposures may be greater in children than adults [18,19,20,21,22]. Other studies have shown that broadcast application of flea control products inside the home can affect human occupants [23]. In addition, it has been demonstrated that pets can bring pesticides into the home after playing in yards that have been treated to control pests and thus, presents another means of exposure to pesticides for children [24,25]. To minimize these potential hazards, children and adults should wash their hands after petting or playing with their pets. Pets should not be allowed to play in lawns treated with pesticides until the product has degraded (time varies depending on the pesticide). Rooms in homes treated with broadcast application of flea-control products should be isolated and ventilated after application of the product according to its label’s directions.

Some of the main contributing factors for pesticide exposure and acute illness or injury events included improper storage of pesticides within reach of children; inappropriate use of a pesticide according to a product’s information label; and application of pesticides manually, through a pressurized can, or through a trigger pump. Children can be primarily exposed to pyrethroid (a type of insecticide)-containing products from dermal exposure with the potential for unintended ingestion when skin lotions or shampoos containing permethrin are used to treat lice or scabies [26]. As almost all of the events reported in this study were unintended, interventions to reduce acute illness/injury from pesticide exposure need to target different age groups of children. For instance, educational intervention may be necessary for older groups of children, whereas, for younger children, minimizing the application of pesticide products around areas where these children play or crawl may be more appropriate.

### Limitations

Data available from poison centers for pesticide exposure events tend to be an underestimate of exposures, often limited to those aware of the consultation service offered by the poison center. Our data lacks information for: race; ethnicity; a family’s socioeconomic status or education level; parents’ occupations for potential take-home exposures of pesticides; quantity of a pesticide applied; and quantity of pesticide absorbed through testing of pesticide concentrations in the body.

## 5. Conclusions

Pesticide exposure events among children can cause serious illness or injury, even death. The findings from this study provide insight into the potential factors contributing to the occurrence of these exposure events and acute illness/injury among children. To minimize pesticide exposure among children, parents need to ensure pesticides are placed in locked cabinets, if stored indoors, or away from the reach of children such as at a nearby outdoor location. Parents also need to ensure adequate ventilation in an indoor room after the application of a pesticide, and verify that pesticide applications from equipment do not leak or spill on surfaces where children are crawling or moving around on floors.
